# Ewastools: Infinium Human Methylation BeadChip pipeline for population epigenetics integrated into Galaxy

**DOI:** 10.1093/gigascience/giaa049

**Published:** 2020-05-13

**Authors:** Katarzyna Murat, Björn Grüning, Paulina Wiktoria Poterlowicz, Gillian Westgate, Desmond J Tobin, Krzysztof Poterlowicz

**Affiliations:** 1 Center for Skin Sciences, University of Bradford, Richmond Road, Bradford BD7 1DP, UK; 2 Freiburg Galaxy Team, University of Freiburg, Fahnenbergplatz, 79085 Freiburg im Breisgau, Germany; 3 Hull York Medical School, University of York, University Rd, York Y010 5DD, UK; 4 The Charles Institute for Dermatology, Belfield, School of Medicine, University College Dublin, Ireland

**Keywords:** Infinium Human Methylation BeadChip, epigenome-wide association studies (EWAS), DNA methylation, Galaxy Project, pipeline, sequence analysis

## Abstract

**Background:**

Infinium Human Methylation BeadChip is an array platform for complex evaluation of DNA methylation at an individual CpG locus in the human genome based on Illumina’s bead technology and is one of the most common techniques used in epigenome-wide association studies. Finding associations between epigenetic variation and phenotype is a significant challenge in biomedical research. The newest version, HumanMethylationEPIC, quantifies the DNA methylation level of 850,000 CpG sites, while the previous versions, HumanMethylation450 and HumanMethylation27, measured >450,000 and 27,000 loci, respectively. Although a number of bioinformatics tools have been developed to analyse this assay, they require some programming skills and experience in order to be usable.

**Results:**

We have developed a pipeline for the Galaxy platform for those without experience aimed at DNA methylation analysis using the Infinium Human Methylation BeadChip. Our tool is integrated into Galaxy (http://galaxyproject.org), a web-based platform. This allows users to analyse data from the Infinium Human Methylation BeadChip in the easiest possible way.

**Conclusions:**

The pipeline provides a group of integrated analytical methods wrapped into an easy-to-use interface. Our tool is available from the Galaxy ToolShed, GitHub repository, and also as a Docker image. The aim of this project is to make Infinium Human Methylation BeadChip analysis more flexible and accessible to everyone.

## Background

Over the past several years comprehensive sequencing datasets have been generated, allowing analysis of genome-wide activity in cohorts of different individuals to be increasingly available. Infinium Human Methylation BeadChip requires only a few days to produce methylome profiles of human samples with a low sample input requirement (as low as 500 ng of genomic DNA) for the starting material [1]. Studies performed recently have identified variation naturally occurring in the genome associated with disease risk and prognosis, including tumour pathogenesis [2]. This raised interest in the concept of epigenome-wide association studies (EWAS). The term “epigenome” means “on top of the genome” and refers to specific changes in genome regulatory activity occurring in response to environmental stimuli [3]. Epigenetic modifications do not change the underlying DNA sequence but can cause multiple changes in gene expression and cellular function [4]. In humans, DNA methylation occurs by attaching a methyl group to the cytosine residue. This has been suggested as a suppressor of gene expression [5]. Multiple methods for DNA methylation analysis were developed, including PCR and pyrosequencing of bisulfite converted DNA, dedicated to studying a small number of methylation sites across a number of samples [6]. Assays like whole-genome bisulfite sequencing and reduced representation bisulfite sequencing allow global quantification of DNA methylation levels. However, running this type of analysis for a larger number of samples can be prohibitively laborious and expensive [7]. The Infinium Human Methylation BeadChip [1] offers unprecedented applicability and affordability owinng to the low costs of reagents, short time of processing, high accuracy, and low input DNA requirements. It determines quantitative array-based methylation measurements at the single-CpG-site level of >850,000 loci [8], covering most of the promoters and also numerous other loci. This makes this assay suitable for systematic investigation of methylation changes in normal and diseased cells [3]. As such it has become one of the most comprehensive solutions on the market [9]. However, Illumina commercial software generates additional costs and is not suitable for everyone. Therefore there is a need to create freely available software able to perform comprehensive analysis including quality control, normalization, and detection of differentially methylated regions (DMRs) [9]. Open source software packages (e.g., DMRcate [10], Minfi [11], ChAMP [12], methylumi [13], RnBeads [14]) require high-performance computational hardware as well as command line experience in order to run the analysis. This is why one of the aims of the our Infinium Human Methylation BeadChip pipeline was to set and implement these methods into a user-friendly environment. The tool has been developed to provide users with an enhanced understanding of the Infinium Human Methylation BeadChip analysis. The workflow includes methods for preprocessing with a stratified quantile normalization preprocess. It includes quantile or extended implementation of the functional normalization preprocess Funnorm with unwanted variation removal, sample-specific quality assessment, and methodology for calling DMR and differentially methylated position (DMP) detection. Scripts were combined and published on the web-based platform Galaxy, a graphical interface with tools and ready-to-run workflows providing a solution for non-programmer scientists to analyse their data and share their experience with others [15]. Configuration files are publicly shared on our GitHub repository [16], with code and dependency settings also available to download and install via the Galaxy ToolShed [17]. Our tool was created and tested using Planemo [18], an integrated workspace for Galaxy tool development with a default configuration and shed tool set-up available via Docker (operating system–level virtualization) [16].

## Tool Description

**Figure 1:**
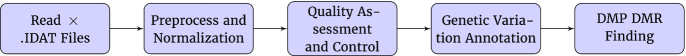
Simplified workflow for analysing epigenetics data.

**Figure 2:**
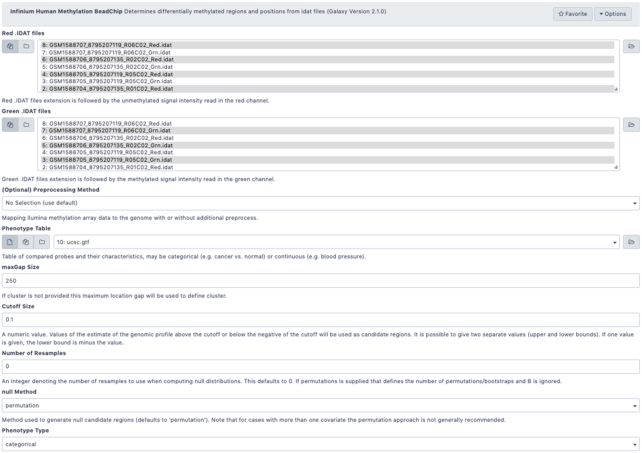
Screenshot from the Galaxy interface, showing Infinium Human Methylation BeadChip workflow as discussed in the case study section.

The workflow combines 5 main steps (see Fig. 1), starting with raw intensity data loading (/.idat) and then preprocessing and optional normalization of the data. The next quality control step performs an additional sample check to remove low-quality data, which normalization cannot detect. The workflow gives the user the opportunity to perform any of these preparation and data-cleaning steps, including the next highly recommended genetic variation annotation step resulting in single-nucleotide polymorphism (SNP) identification and removal. Finally, the dataset generated through all of these steps can be used to find DMPs and DMRs with respect to a phenotype covariate. All the steps, as well as simple preparation and analysis options, are shown in Fig. 2 and explained in detail below.

### Data loading

The Infinium Human Methylation BeadChip assay interrogates fluorescent signals (green and red) from the methylated and unmethylated sites into binary values that can be read directly as IDAT files [1]. Illumina’s GenomeStudio (GenomeStudio, RRID:SCR_010973) solution converts the data into plain-text ASCII files, losing a large amount of information during this process [19]. To prevent this kind of data loss we introduced an R-based .IDAT file-loading method, which is a combination of illuminaio readIDAT and minfi RGChannelSet functions. It reads intensity information from both treatment and control data and based on this it builds up a specific joined dataset.

### Preprocessing and normalization

Green and red channel signals from .IDAT files can be converted into methylated and unmethylated signals assigned to methylation levels or β values. β are built in RatioSet object, and they estimate the methylation level using channel ratios in a range between 0 and 1, with 0 being unmethylated and 1 being fully methylated [19]. However, these 2 classes can also be preprocessed and normalized with 2 methods available [19]. Preprocess Quantile implements stratified quantile normalization preprocessing and is supported for small changes such as in 1-type samples, e.g., blood datasets. In contrast, preprocess Funnorm is aimed at global biological differences such as healthy and occurred datasets with different tissue and cell types. This is called the “between-array normalization method” and removes unwanted variation [19]. In addition unwanted probes containing either an SNP at the CpG interrogation or at the single-nucleotide extension can be removed (recommended) [19].

### Quality assessment and control

Data quality assurance is an important step in Infinium Human Methylation BeadChip analysis. The quality control function extracts and plots the data frame with 2 columns mMed and uMed, which are the medians of MethylSet signals (Meth and Unmeth). Comparing these against one another allows users to detect and remove low-quality samples that normalization cannot correct [11].

### Annotating probes affected by genetic variation

SNP regions may affect results of downstream analysis. The Remove SNPs step returns data frames containing the SNP information of unwanted probes and removes them from the dataset [19].

### DMP and DMR identification

The main goal of the Infinium Human Methylation BeadChip tool is to simplify the way differentially methylated locus sites are detected. The workflow contains a function detecting DMPs with respect to the phenotype covariate, and a method for finding DMRs [11]. DMRs can be tracked using a bump-hunting algorithm. The algorithm first implements a *t*-statistic at each methylated locus location, with optional smoothing, then groups probes into clusters with a maximum location gap and a cut-off size to refer the lowest possible value of genomic profile hunted by our tool [20].

### Functional annotation and visualization

**Figure 3:**
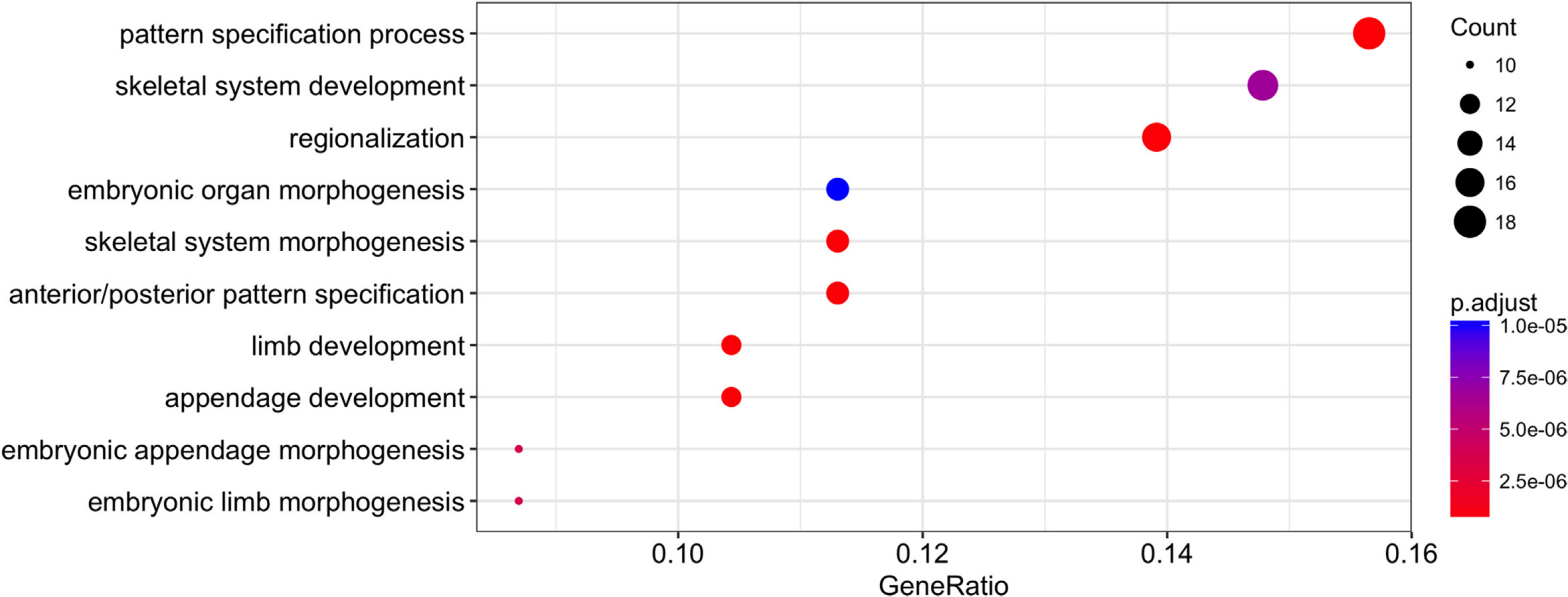
Functional annotation of DMRs found in melanoma biopsies before and after MAPKi treatment.

In addition to downstream analysis, users can access annotations provided via Illumina by ChIPpeakAnno (ChIPpeakAnno, RRID:SCR_012828) annoPeaks tool [19] or perform additional functional annotations using the Gene Ontology (GO) via Cluster Profiler GO tool. The GO tool provides a very detailed representation of functional relationships between biological processes, molecular function, and cellular components across data [21]. Once specific regions have been chosen, Cluster Profiler GO visualizes enrichment results (see Fig. 3). Many researchers use annotation analysis to characterize the function of genes, which highlights the potential for Galaxy to be a solution for wide-ranging multi-omics research.

### Documentation and training

We have also provided training sessions and interactive tours for user self-learning. The training materials are freely accessible at the Galaxy (Galaxy, RRID:SCR_006281) project Github repository [22]. Such training and tours guide users through an entire analysis. The following steps and notes help users to explore and better understand the concept. Slides and hands-on instruction describe the analysis workflow, and all necessary input files are ready to use via Zenodo [23], as well as a Galaxy Interactive Tour, and a tailor-made Galaxy Docker image for the corresponding data analysis.

### Case study

**Figure 4:**
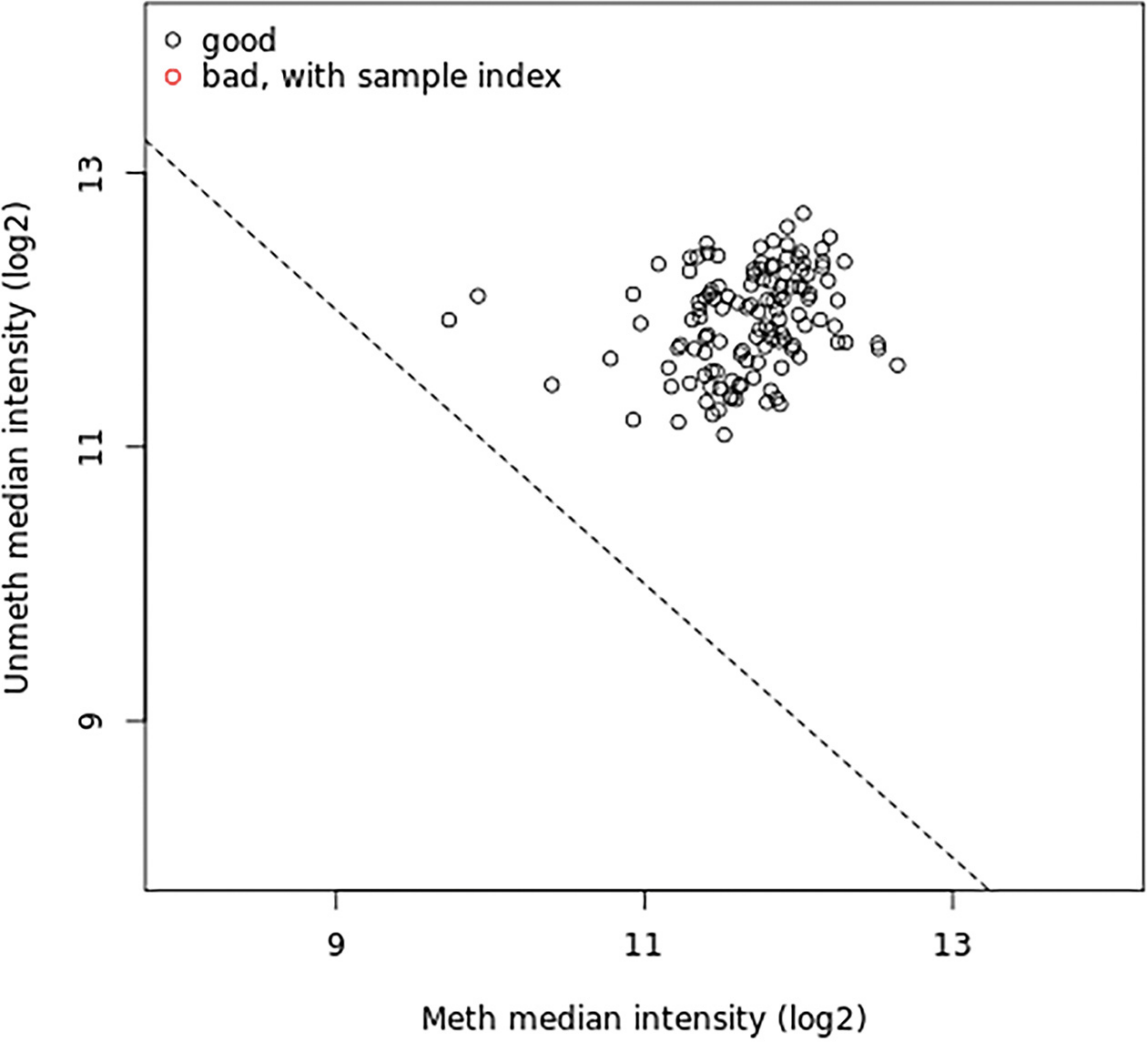
Quality control plot representing median intensity of melanoma in samples before and after MAPKi treatment. The plot compares median total intensity (log2) of the methylated channel (x-axis) and unmethylated channel (y-axis). Bad-quality samples fall under the threshold and are colored red. There is no bad-quality sample in this study

Compared to genetic studies EWAS provides a unique opportunity to study dynamic response to treatment. It has been suggested that DNA methylation is associated with drug resistance [24]. To validate our suite we have performed analysis of differentially methylated regions using publicly available data from the Infinium Human Methylation BeadChip array of melanoma biopsies before and after mitogen-activated protein kinase inhibitor (MAPKi) treatment [25], obtained from the Gene Expression Omnibus (GEO) (GSE65183). Methylation profiling by genome tiling array in melanoma can help us understand how non-genomic and immune changes can have an impact on treatment efficiency and disease progression. Raw image IDAT files were loaded into the Galaxy environment using Data Libraries. EWAS workflow was run on Red and Green dataset collections of patient-matched melanoma tumours biopsied before therapy and during disease progression. The IDAT files, predefined phenotype tables, and up-to-date genome tables (UCSC Main on Human hg19 Methyl450) [16] were used as inputs. To detect poorly performing samples we ran quality diagnostics. The provided samples passed the quality control test (in Fig. 4 ) because they clustered together with higher median intensities, confirming their good quality [19]. Differentially methylated loci were identified using single-probe analysis implemented by our tool with the following parameters: phenotype set as “categorical” and qCutoff size set to 0.05. The bump-hunting algorithm was applied to identify DMRs with maximum location gap parameter set to 250, genomic profile above the cut-off equal to 0.1, number of resamples set to 0, and null method set to “permutation and verbose equal FALSE,” which means that no additional progress information will be printed. Differentially Methylated Regions and Positions revealed the need for further investigation of tissue diversity in response to environmental changes [26]. The nearest transcription start sites found in the gene set can be listed as follows: PITX1, SFRP2, MSX1, MIR21, AXIN2, GREM1, WT1, CBX2, HCK, GTSE1, SNCG, PDPN, PDGFRA, NAF1, FGF5, FOXE1, THBS1, DLK1, and HOX gene family. The results of the re-analysis are available in the GitHub repository [27].

### Important findings

Although hypermethylated genes identified by “EWAS-suite” have been previously associated with cancer, this is the first time a link between them and MAPKi treatment resistance is reported. This data demonstrates the presence of platelet-derived growth factor receptor (PDGFR), which is suggested to be responsible for RAS/MAPK pathway signaling. Trough activation may regulate the MAPKi mechanism in non-responsive tumours. The methylation regulation of this altered status of PDGFR requires additional studies [25]. The PITX1 suppressor gene was found as one of the factors decreasing gene expression in human cutaneous malignant melanoma and might contribute to progression and resistance via promoting cell proliferative activity [28]. It has been found that the homeodomain transcription factor MSX1 and the CBX2 polycomb group protein are likely to be treatment resistance factors and are reported as downregulated and inactivated in melanoma tumours [29]. Previous published studies are limited to local surveys and serial biopsies. Thus, the stimulus of innate or acquired MAPKi resistance may be linked to epigenetics. GO annotation provides information regarding the function of genes [30]. GO analysis identified the pattern specification process (GO:0007389), skeletal system development (GO:0001501), and regionalization (GO:000300) as significantly overrepresented categories within the above DMRs, suggesting that melanoma MAPKi resistance could be related to the cells' developmental process within specific environments.

## Conclusion

With the rapidly increasing volume of epigenetics data available, computer-based analysis of heritable changes in gene expression becomes more and more feasible. Many genome-wide epigenetics studies have focused on generation of data, with data interpretation now being the challenge. Risk evaluation, disease management, and novel therapeutic development are prompting researchers to find new bioinformatic frameworks and approaches. In this regard we propose a user-friendly tool suite available via Galaxy platform. Ewastools allows life scientists to run complex epigenetics analysis [16]. The case study presented provides a tangible example of how population epigenetics analysis can provide additional insights into melanoma therapeutic resistance.

## Availability of Source Code and Requirements

- Project name: Ewastools: Infinium Human Methylation BeadChip pipeline for population epigenetics integrated into Galaxy
- Project home page: https://github.com/kpbioteam/ewas_galaxy
- Operating system(s): Linux (recommended), Mac
- Programming language: R programming language (version 3.3.2, x86 64bit)
- Other requirements: Galaxy [22], Docker [31]
- License: MIT License
- biotoolsID identifier: https://bio.tools/ewastools
- RRID:SCR_018085

## Availability of Supporting Data and Materials

The test dataset in this article is available in the GEO database under accession GSE65186. The results of the re-analysis of the GSE65186 dataset are available in the GitHub repository (https://github.com/kpbioteam/ewastools-case_study). All tools described here are available in the Galaxy ToolShed (https://toolshed.g2.bx.psu.edu). The Dockerfile required to automatically deploy the pre-built Docker image is available at https://galaxyproject.org/use/ewas-galaxy/. Archival snapshots of the code are available in the *GigaScience* GigaDB repository [32].

## Abbreviations

DMP: differentially methylated position; DMR: differentially methylated region; EWAS: epigenome-wide association study; GEO: Gene Expression Omnibus; GO: Gene Ontology; MAPKi: mitogen-activated protein kinase inhibitor; PDGFR: platelet-derived growth factor receptor; SNP: single-nucleotide polymorphism; UCSC: University of California Santa Cruz.

## Competing Interests

The authors declare that they have no competing interests.

## Authors' Contributions

K.P. conceived and designed the study, K.P., K.M. and B.G. developed the software, K.P., K.M., P.W.P. and B.G. did testing, K.P., K.M. and P.W.P. performed the analyses, K.P, K.M, D.J.T and G.W. provided biological interpretation. All authors wrote the manuscript. All authors read and approved the final manuscript.

## Supplementary Material

giaa049_GIGA-D-19-00088_Original_Submission

giaa049_GIGA-D-19-00088_Revision_1

giaa049_GIGA-D-19-00088_Revision_2

giaa049_Response_to_Reviewer_Comments_Original_Submission

giaa049_Response_to_Reviewer_Comments_Revision_1

giaa049_Reviewer_1_Report_Original_Submission Mallory Freeberg, Ph.D. -- 4/10/2019 Reviewed

giaa049_Reviewer_1_Report_Revision_1 Mallory Freeberg, Ph.D. -- 3/14/2020 Reviewed

giaa049_Reviewer_2_Report_Original_Submission Daniel Blankenberg -- 10/28/2019 Reviewed
