# Supplementary material for: Ewastools: Infinium Human Methylation BeadChip pipeline for population epigenetics integrated into Galaxy
Source: Gigascience. 2020 May 13;9(5):giaa049. doi: 10.1093/gigascience/giaa049 (PMC7219210; doi:10.1093/gigascience/giaa049)
Supplement: giaa049_GIGA-D-19-00088_Revision_1 [file giaa049_giga-d-19-00088_revision_1.pdf]

GigaScience

Ewastools: Infinium Human Methylation BeadChip pipeline for population epigenetics integrated into Galaxy

--Manuscript Draft--

**Manuscript Number:**	GIGA-D-19-00088R1
**Full Title:**	Ewastools: Infinium Human Methylation BeadChip pipeline for population epigenetics integrated into Galaxy
**Article Type:**	Technical Note
**Funding Information:**	
**Abstract:**	Background, Infinium Human Methylation BeadChip is an array platform for complex evaluation of DNA methylation at an individual CpG loci in the human genome based on Illumina's bead technology and It is one of the most common techniques used in epigenome-wide association studies (EWAS). Finding associations between epigenetic variation and phenotype is a significant challenge in biomedical research. The newest version, Human Methylation EPIC, quantifies the DNA methylation level of 850k CpG sites, while the previous versions, HumanMethylation450 and HumanMethylation27, measured over 450k and 27k loci, respectively. Although a number of bioinformatics tools have been developed to analyse this assay, they require some programming skills and experience in order to be usable .Results, We have developed a pipeline for the Galaxy platform for those without experience aimed at DNA methylation analysis using the Infinium Human Methylation BeadChip. Our tool is integrated into Galaxy (http://galaxyproject.org), a web based platform. This allows users to analyse data from the Infinium Human Methylation BeadChip in the easiest possible way. Conclusions, The pipeline provides a group of integrated analytical methods wrapped into an easy to use interface. Our tool is available from the Galaxy toolshed, GitHub repository and also as a Docker image. The aim of this project is to make Infinium Human Methylation BeadChip analysis more flexible and accessible to everyone.
**Corresponding Author:**	Krzysztof Poterlowicz<br>University of Bradford<br>Bradford, Bradford UNITED KINGDOM
**Corresponding Author Secondary Information:**	
**Corresponding Author's Institution:**	University of Bradford
**Corresponding Author's Secondary Institution:**	
**First Author:**	Katarzyna Murat
**First Author Secondary Information:**	
**Order of Authors:**	Katarzyna Murat
	Björn Grüning
	Paulina Wiktoria Poterlowicz
	Gillian Westgate
	Desmond J Tobin
	Krzysztof Poterlowicz
**Order of Authors Secondary Information:**	
**Response to Reviewers:**	We would like to thank the reviewers and the editors for their careful assessment of the manuscript and their detailed and constructive comments. We have extended the manuscript, clarified the methodologies and performed additional new experiments. Below we provide point-to-point responses with reference to the corresponding modifications of the manuscript. We believe that addressing these comments has produced a stronger manuscript that will hold more value for the scientific community.

Powered by Editorial Manager® and ProduXion Manager® from Aries Systems Corporation

#Editor Comments: In addition, please register any new software application in the bio.tools and SciCrunch.org databases to receive RRID (Research Resource Identification Initiative ID) and biotoolsID identifiers, and include these in your manuscript. This will facilitate tracking, reproducibility and re-use of your tool.

Authors:
Thank you for considering our revised manuscript. We have registered our tool in both bio.tools and in SciCrunch.org, and have provided the identifiers in our manuscript: biotoolsID identifier: biotools:ewastools, RRID: SCR_018085

Reviewer #1 Major concern:
The "Potential implications" section is confusing. It reads more of a use case or a proof of concept illustration of the value of the EWAS-Galaxy tools suite, rather than a section that discusses the "potential implications" for EWAS-Galaxy. I do like the idea of providing a use case, but I suggest renaming this section. I also suggest adding a little more background about the dataset being tested. Why was it chosen (the fact that there is "interest in skin cancer biomarker identification" doesn't seem like enough of a reason)? The dataset is published, which leads me to believe the authors are doing a re-analysis of the study. How do their results of identifying a set of DMRs/DMPs near transcription start sites and enhancers of the listed genes compare to what the original authors of the study found? I would love to see this use case expanded as I believe the goal is to highlight that EWAS-Galaxy can analyze (re-analyze?) methylation array data to drive hypothesis generation, which is an important point to make.
Authors:
We sincerely thank the reviewer for their thoughtful comments and inspections of our submission. We have renamed the section as suggested and provided more background about the dataset being tested (" …. Compared to genetic studies EWAS provides a unique opportunity to study dynamic response to treatment. It has been suggested that DNA methylation is associated with drug resistance.To validate our suite we have performed analysis of differentially-methylated regions using publicly available data from the Infinium Human Methylation BeadChip array of melanoma biopsies pre and post MAPKi treatment…."). The authors cannot agree more with the reviewer that it would be interesting to compare our results with what the original authors of the study found. Unfortunately the original study only provides a selected fragment of the results of the data analysis. Therefore we have created a dedicated GitHub repository https://github.com/kpbioteam/ewastools-case_study with the results of re-analysis of the original dataset.

Minor concerns:
Reviewer #1 comment#1
In the Background section the authors mention multiple open source software packages for analyzing methylation assay data (page 2, line 25). It appears that only Minfi tools made it into EWAS-Galaxy. It would be great if the authors mentioned whether there is ongoing work to incorporate these additional tools into EWAS-Galaxy or why only Minfi tools were included.
Authors:
We thank the reviewer for this comment. For now, we have focused on the implementation of the Minfi tools in the galaxy. We are happy to add additional tools if requested by users.
Reviewer #1 comment 2
The sentence starting "The tool suite includes methods…" on page 2, line 31 is weirdly worded. Bolded names of the tools are inserted into the sentence in a way that makes the sentence hard to read. The same weird pattern is present on page 2 line 19. I would suggest re-wording these sentences to match the wording in the "Preprocessing and Normalization" and "Quality Assessment and Control" sections (where the bolded tool names make sense in the sentences).
Authors:
We agreed with this comment and changed the sentences accordingly
Reviewer #1 comment 3
There is mention of Illumina Genome Studio (page 2, line 20) before saying what it is (in Data Loading section). There is mention of Planemo (page 2, line 44) without mentioning or citing what it is. I would suggest describing these (and any other specialty) terms the first time they are mentioned.

Powered by Editorial Manager® and ProduXion Manager® from Aries Systems Corporation

Authors:
We agreed with this comment and changed the sentences accordingly
Reviewer #1 comment 4
I am unsure whether mentions of "Illumina Methylation Assay" (page 2, line 11), "450k assay" (page 2, line 14), "Infinium Methylation Assay" (page 2, line 48), and "Illumina 450k Methylation" (page 3, line 38) are all referring to the same assay type. I would suggest being consistent with naming or explicit about whether the different terms are the same assay type.
Authors:
We agreed with this comment and changed the sentences accordingly
Reviewer #1 comment 5
It is unclear what "bad, with sample index" means in the Figure 3 graph legend. Please clarify.
Authors:
We agreed with this comment and changed the sentences accordingly
Reviewer #1 comment 6
There is duplication of spelling out terms followed by the abbreviation in parentheses. In one example, "differentially-methylated regions (DMRs)" can be found three times in the text (page 2 line 62, page 3 line 35, and page 3 line 58). As per author instructions: "If abbreviations are used in the text they should be defined in the text at first use".
Authors:
We thank the reviewer for their careful inspection and modified accordingly
Reviewer #1 comment 7
An Abbreviations section is missing from the manuscript. As per author instructions: "a list of abbreviations should be provided in alphabetical order.".
Authors:
We agreed with this comment and provided list of abbreviations
Reviewer #1 comment 8
Figure numbering appears out of order. Figure 5 is called out before Figure 3. I do not see a call out to Figure 4. I also am not sure what conclusion I am supposed to draw from Figure 4. I suggest numbering and ordering the figures as they appear in the text and providing an explanation of what Figure 4 is showing.
Authors:
We thank the reviewer for pointing out this inconsistency, which has now been corrected.
Reviewer #1 comment 9
The Availability and requirements section is formatted strangely, and the section header includes "(Availability of source code and requirements (optional, if code is present))", which looks like it was copied from the author instructions and not removed. Please check formatting.
Authors:
We agreed with this comment and changed the section title and formatting accordingly
Reviewer #1 comment 10
There is mixed usage of US and UK English spelling (e.g. normalization and normalisation). Please standardize.
Authors:
We agreed with this comment and changed the spelling accordingly
Reviewer #2: Formatted review attached as PDF. Raw text is below.

Major concerns
Article Text
Reviewer #2 comment 1
This article, and especially the title, seem to indicate that the described tool suite is generally applicable to most/all population epigenetic study analysis.
However, the current implementation of this tool suite is limited to only handling Illumina Infinium methylation arrays, in particular the IlluminaHumanMethylation450 ('450k') array.
If your population epigenetic datasets are not from this specific technology iteration, then you cannot use these Galaxy tool implementation to perform EWAS.
Either the article and title should be adjusted to accurately reflect the abilities of the described tools, or the tools should be updated to handle additional, including non-illumina methylation array, dataset types. This tool suite is currently several wrappers around minfi functions, that has been restricted to working with IlluminaHumanMethylation450kanno.ilmn12.hg19.

Authors:
We thank the reviewer for the comment. We have extended the tool suite to support: HumanMethylationEPIC and HumanMethylation27 methylation arrays. We fully understand the concerns of the reviewer around usability of the tool suite for population epigenetic datasets that are not from Illumina Infinium methylation arrays. However, at this moment the EWAS studies are predominantly generated using Illumina Infinium methylation arrays. EWAS Atlas (https://bigd.big.ac.cn/ewas) is one of the most comprehensive knowledge base of epigenome wide association studies and among the 1160 studies reported there we haven't found a single one that used a platform different than HumanMethylationEPIC ('850k') or IlluminaHumanMethylation450 ('450k') [accessed on 20/02/2020] . We are happy to add additional tools if requested by users.
We have also adjusted the article title to "Ewastools: Infinium Human Methylation BeadChippipeline for population epigenetics integrated into Galaxy"

Reviewer #2 comment 2
Confirm that all tools e.g. listed in Table 1 are available in the ToolShed, Docker image, etc. (for example, the minfi_getanno does not exist, etc.)
Authors
We thank the reviewer for their careful inspection. We have not included Table 1 in the reviewed version of the manuscript as addressing one of the reviewer's comment below, the recent version of the suite consists a single 'tool' with multiple functions.

Software
Reviewer #2 comment 3
Interoperability concerns. Stated purpose of toolset is to increase accessibility to EWAS methods, but toolset is very specific and not interoperable. 'Rdata' datatype is used as intermediate and end-pint datasets. Potential security concerns introduced by tools that consume untrusted serialized datasets, e.g. 'rdata' Rdata datasets cannot be used as inputs by standard Galaxy tools, so, essentially user gets 'locked in' to this pipeline implementation.
All outputs are a base 'rdata' datatype, despite containing different objects. This allows improper input/output dataset mixing. Hierarchical datatypes should be used.
Really, an intermediate filetype that is usable by other standard Galaxy tools (e.g. tabular files) would be advisable. At the very least, there should be tools to export to and from these specific binary types and more generalized formats.
Authors:

We agree with the reviewer and have modified the toolset so 'Rdata' datatype is no longer used.

Reviewer #2 comment 4
Majority of tools have an input for datasets and output of another. The interfaces do not allow configuring other options to the various functions.
Because intermediate datasets are rdata, and there are several steps that are just input file → output file (no other configurable), I have questions about the need to actually have them as separate steps. Does it make sense to have a 'tool' consist of multiple function calls instead?

Authors:
We agree with the reviewer and the recent version of the suite consists of a single tool with multiple functions.

Reviewer #2 comment 5
Minfi Map to Genome map to genome tool should assign dbkey to output dataset metadata in Galaxy
Tool should also allow selecting from a list of genomes/annotations
Would allow the other illumina arrays supported by minfi to work in this toolset
Authors:
We thank the reviewer for the comment. The recent version of the suite allows selecting different types of illumine arrays (>Genome Table option). We removed Minfi Map to Genome map to genome tool as the suite consists of a single tool with multiple functions.

Powered by Editorial Manager® and ProduXion Manager® from Aries Systems Corporation

	Reviewer #2 comment 6<br>A "Galaxy Interactive Tour" is described as being available for the EWAS tools, but the tour.yaml file contains only a name and a description and not any actual tour content. (https://github.com/galaxyproject/training-material/blob/master/topics/epigenetics/tutorials/ewas-suite/tours/tour.yaml)<br><br>Authors:<br>We thank the reviewer for pointing out this inconsistency, which has now been corrected in the recent version of the training material https://github.com/galaxyproject/training-material/pull/1778<br><br><br>Reviewer #2 comment 7<br>Add license to https://github.com/kpbioteam/ewas_galaxy<br>Authors:<br>We thank the reviewer for the comment. We updated as requested.<br><br>Minor concerns<br>Reviewer #2 comment 8<br>Docker container is using Galaxy 18.05 and the pre-loaded EWAS tools are out-of-date Should update to a newer version of Galaxy and update the contained EWAS-Galaxy tools. (docker run -it -p 8080:80 --rm kpbioteam/galaxy-ewas)<br>Logged in as an admin, and updated tools from ToolShed for the purpose of this review<br>Output of minfi dmr and minfi dmr tools creates an 'interval' type dataset. But these outputs have non-commented header lines, and cannot be displayed at UCSC as shown in tutorial without additional manual processing. This should be fixed at the tool output.<br>Authors:<br>We agree with the reviewer and have updated the docker container to Galaxy 19:09 and updated the contained tools from ToolShed. We have modified the tool to output ‘dmr’ and ‘dmp’ datasets as a ‘bed’ type<br>Reviewer #2 comment 9<br>The "Minfi Read 450k load .IDAT files" tool requires the datasets in the history to have names that have specific '_Red.idat' and '_Grn.idat' pattern matching. This is not explained in the tool, and if dataset names do not match, no warning or error is displayed to the user. This should be fixed as part of the tool.<br>Authors:<br>We thank the reviewer for the comment. We have explained now in the tool, the requirements for the datasets in the history to have names that have specific '_Red.idat' and '_Grn.idat' pattern matching i.e (“*Inputs*Series of .IDAT files: matching red and green .idat file for each sample on the chip intensity data”). This format follows the standard raw input for Illumina Methylation arrays as each sample have two files: one for red and green channels respectively.<br>Reviewer #2 comment 10<br>Authors:<br>Training material tutorial (https://training.galaxyproject.org/training-material/topics/epigenetics/tutorials/ewas-suite/tutorial.htm) has several errors, with described tool configurations/interfaces lacking the declared options, etc (e.g. minfi dmr tool, etc).<br>Also some missing steps (e.g. removing first lines of 'fake' interval files). Recommend walking through tutorial again and confirming all steps are listed and clear<br><br>We thank the reviewer for their careful inspection, which has now been corrected in the recent version of the training material https://github.com/galaxyproject/training-material/pull/1778.
**Additional Information:**	
**Question**	**Response**
Are you submitting this manuscript to a special series or article collection?	No
**Experimental design and statistics**	Yes

Full details of the experimental design and statistical methods used should be given in the Methods section, as detailed in our Minimum Standards Reporting Checklist. Information essential to interpreting the data presented should be made available in the figure legends.<br><br>Have you included all the information requested in your manuscript?	
**Resources**<br><br>A description of all resources used, including antibodies, cell lines, animals and software tools, with enough information to allow them to be uniquely identified, should be included in the Methods section. Authors are strongly encouraged to cite Research Resource Identifiers (RRIDs) for antibodies, model organisms and tools, where possible.<br><br>Have you included the information requested as detailed in our Minimum Standards Reporting Checklist?	Yes
**Availability of data and materials**<br><br>All datasets and code on which the conclusions of the paper rely must be either included in your submission or deposited in publicly available repositories (where available and ethically appropriate), referencing such data using a unique identifier in the references and in the "Availability of Data and Materials" section of your manuscript.<br><br>Have you have met the above requirement as detailed in our Minimum Standards Reporting Checklist?	Yes

LaTeX - Main Document (TeX file) Click here to access/download;LaTeX - Main Document (TeX file);main.tex

OXFORD (GIGA)^n SCIENCE

GigaScience, 2018, 1–6

doi: xx.xxxx/xxxx
Manuscript in Preparation
Technical note

TECHNICAL NOTE

Ewastools: Infinium Human Methylation BeadChip pipeline for population epigenetics integrated into Galaxy

Katarzyna Murat[1], Björn Grüning[2], Paulina Wiktoria Poterlowicz[3], Gillian Westgate[1], Desmond J Tobin[4,1] and Krzysztof Poterlowicz[1,✉]

[1]Center for Skin Sciences, University of Bradford,Bradford, BD7 1DP, United Kingdom and [2]Freiburg Galaxy Team, University of Freiburg, Fahnenbergplatz, 79085 Freiburg im Breisgau, Germany and [3]Hull York Medical School, University of York, York, Y010 5DD, United Kingdom and [4]The Charles Institute for Dermatology, School of Medicine, University College , Dublin, Ireland

✉k.poterlowicz1@bradford.ac.uk

Abstract

Background, Infinium Human Methylation BeadChip is an array platform for complex evaluation of DNA methylation at an individual CpG loci in the human genome based on Illumina's bead technology and It is one of the most common techniques used in epigenome-wide association studies (EWAS). Finding associations between epigenetic variation and phenotype is a significant challenge in biomedical research. The newest version, HumanMethylationEPIC, quantifies the DNA methylation level of 850k CpG sites, while the previous versions, HumanMethylation450 and HumanMethylation27, measured over 450k and 27k loci, respectively. Although a number of bioinformatics tools have been developed to analyse this assay, they require some programming skills and experience in order to be usable. **Results**, We have developed a pipeline for the Galaxy platform for those without experience aimed at DNA methylation analysis using the Infinium Human Methylation BeadChip. Our tool is integrated into Galaxy (http://galaxyproject.org), a web based platform. This allows users to analyse data from the Infinium Human Methylation BeadChip in the easiest possible way. **Conclusions**, The pipeline provides a group of integrated analytical methods wrapped into an easy to use interface. Our tool is available from the Galaxy toolshed, GitHub repository and also as a Docker image. The aim of this project is to make Infinium Human Methylation BeadChip analysis more flexible and accessible to everyone.

Key words: Infinium Human Methylation BeadChip; Epigenome-Wide Association Studies (EWAS); DNA methylation; Galaxy Project; Pipeline; Sequence analysis

Background

Over the last several years comprehensive sequencing data sets have been generated, allowing analysis of genome-wide activity in cohorts of different individuals to be increasingly available. Infinium Human Methylation BeadChip requires only a few days to produce methylome profiles of human samples with low sample input requirement (as low as 500 ng of genomic DNA) as the starting material [1]. Studies performed recently have identified variation naturally occurring in the genome associated with disease risk and prognosis, including tumour pathogenesis [2]. This raised interest in the concept of epigenome-wide association studies (EWAS). The term Epigenome means "on top of" the genome and refers to specific changes in genome regulatory activity occurring in response to environmental stimuli [3]. Epigenetic modifications do not

Compiled on: February 24, 2020.
Draft manuscript prepared by the author.

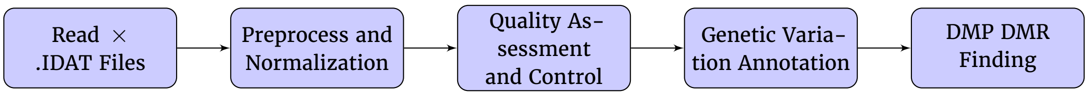

Figure 1. Simplified workflow for analysing epigenetics data

change the underlying DNA sequence, but can cause multiple changes in gene expression and cellular function [4]. In humans, DNA methylation occurs by attaching a methyl group to the cytosine residue. This has been suggested as a suppressor of gene expression [5]. Multiple methods for DNA methylation analysis were developed, including the polymerase chain reaction (PCR) and pyrosequencing of bisulfite converted DNA, dedicated to study a small number of methylation sites across a number of samples [6]. Assays like whole genome bisulfite sequencing (WGBS) and reduced representation bisulfite sequencing (RRBS) allow global quantification of DNA methylation levels. However, running this type of analysis for a larger number of samples can be prohibitively laborious and expensive [7]. The Infinium Human Methylation BeadChip [1] offers unprecedented applicability and affordability due to the low costs of reagents, short time of processing, high accuracy and low input DNA requirements. It determines quantitative array-based methylation measurements at the single-CpG-site level of over 850k loci [8] covering most of the promoters and also numerous other loci. The makes this assay suitable for systematic investigation of methylation changes in normal and diseased cells [3]. As such it has become one of the most comprehensive solutions on the market [9]. However, Illumina commercial software generates additional costs and it is not suitable for everyone. Therefore there is a need to create freely available software able to perform comprehensive analysis including quality control, normalisation and detection of differential-methylated regions [9]. Open-source software packages (e.g. DMRcate [10], Minfi [11], ChAMP [12], methylumi [13], RnBeads [14]) require high performance computational hardware as well as command line experience in order to run the analysis. This is why one of aims of the our Infinium Human Methylation BeadChip pipeline was to set and implement these methods into a user-friendly environment. The tool have been developed to provide users with an enhanced understanding of the Infinium Human Methylation BeadChip analysis. Workflow includes methods for preprocessing with stratified quantile normalisation preprocess. Quantile or extended implementation of functional normalisation preprocess Funnorm with unwanted variation removal, sample specific quality assessment and methodology for calling differentially-methylated regions and sites (DMR) and positions detection (DMP). Scripts were combined and published on the web based platform - Galaxy, a graphical interface with tools, ready to run workflows providing a solution for non-programmer scientists to analyse their data and share their experience with others [15]. Configuration files are publicly shared on our GitHub repository [16] with code and dependency settings also available to download and install via the Galaxy toolshed [17]. Our tool was created and tested using a Planemo [18] an integrated workspace for Galaxy tools development with a default configuration and shed tool setup available via Docker (operating-system-level virtualization) [16].

Tools Description

The workflow combines 5 main steps (see Figure 1), starting with raw intensity data loading (/.idat) and then preprocessing and optional normalisation of the data. The next quality control step performs an additional sample check to remove low-quality data, which normalisation cannot detect. The workflow gives the user the opportunity to perform any of these preparation and data cleaning steps, including the next highly recommended genetic variation annotation step resulting in single nucleotide polymorphism identification and removal. Finally, the dataset generated through all of these steps can be used to find DMPs and regions DMRs with respect to a phenotype covariate. All the steps as well as single preparation and analysis options are shown in Figure 2 and explained in detail below.

Data Loading

The Infinium Human Methylation BeadChip assay interrogates fluorescent signals (qreen and red) from the methylated and unmethylated sites into binary values which can be read directly as IDAT files [1]. Illumina's GenomeStudio solution converts the data into plain-text ASCII files losing a large amount of information during this process [19]. To prevent this kind of data loss we introduced an R based .IDAT files loading method which is a combination of illuminaio readIDAT and minfi RGChannelSet functions. It reads intensity information from both treatment and control data and based on this it builds up a specific joined data set.

Preprocessing and Normalisation

Green and red channel signals from .IDAT files can be converted into methylated and unmethylated signals assigned to methylation levels or Beta values. Betas are build in RatioSet object, and estimate the methylation level using channel ratios in a range between 0 and 1 with 0 being unmethylated and 1 being fully methylated [19]. However, these two classes can also be preprocessed and normalised with two methods available [19]. Preprocess Quantile implements stratified quantile normalisation preprocessing and is supported for small changes like in one-type samples e.g. blood datasets. In contrast, preprocess Funnorm is aimed at global biological differences such as healthy and occurred datasets with different tissue and cell types. This is called the between-array normalisation method and removes unwanted variation [19]. In addition unwanted probes containing either an SNP at the CpG interrogation or at the single nucleotide extension can be removed (recommended)[19].

Quality Assessment and Control

Data quality assurance is an important step in Infinium Human Methylation BeadChip analysis. The quality control function extracts and plots the data frame with two columns mMed and uMed which are the medians of MethylSet signals (Meth and Unmeth). Comparing these against one another allows users to detect and remove low-quality samples that normalisation cannot correct [11].

Annotating probes affected by genetic variation

Single nucleotide polymorphism (SNP) regions may affect results of downstream analysis. Remove SNPs step returns data frames containing the SNP information of unwanted probes

and removes them from the data set [19].

DMPs and DMRs Identification

The main goal of the Infinium Human Methylation BeadChip tool is to simplify the way differentially-methylated loci sites are detected. The workflow contains a function detecting DMPs with respect to the phenotype covariate, and method for finding DMRs [11]. DMRs can be tracked using a bump hunting algorithm. The algorithm first implements a t-statistic at each methylated loci location, with optional smoothing, then groups probes into clusters with a maximum location gap and a cutoff size to refer the lowest possible value of genomic profile hunted by our tool [20].

Functional Annotation and Visualisation

In addition to downstream analysis, users can access annotations provided via Illumina by ChIPpeakAnno annoPeaks tool [19] or perform additional functional annotations using the Gene Ontology (GO) via Cluster Profiler GO tool. The GO tool provides a very detailed representation of functional relationships between biological processes, molecular function and cellular components across data [21]. Once specific regions have been chosen, Cluster Profiler GO visualises enrichment results (see Figure 4). Many researchers use annotation analysis to characterise the function of genes, which highlights the potential for Galaxy to be a solution for wide-ranging multi-omics research.

Documentation and Training

We have also provided training sessions and interactive tours for user self-learning. The training materials are freely accessible at the Galaxy project Github repository [22]. Such training and tours guide users through an entire analysis. The following steps and notes help users to explore and better understand the concept. Slides and hands-on instruction describes the analysis workflow, all necessary input files are ready-to-use via Zenodo [23], as well as a Galaxy Interactive Tour, and a tailor-made Galaxy Docker image for the corresponding data analysis.

Case Study

Compared to genetic studies EWAS provides a unique opportunity to study dynamic response to treatment. It has been suggested that DNA methylation is associated with drug resistance [24].To validate our suite we have performed analysis of differentially-methylated regions using publicly available data from the Infinium Human Methylation BeadChip array of melanoma biopsies pre and post MAPKi treatment [25], obtained from the Gene Expression Omnibus (GEO) (GSE65183). Methylation profiling by genome tiling array in melanoma can help us understand how non-genomic and immune changes can have an impact on treatment efficiency and disease progression. Raw image IDAT files were loaded into the Galaxy environment using Data Libraries. EWAS workflow was run on Red and Green dataset collections of patient-matched melanoma tumours biopsied before therapy and during disease progression. The IDAT files, pre-defined phenotype tables and up-to-date genome tables (UCSC Main on Human hg19 Methyl450) [16] were used as inputs. In order to detect poorly performing samples we ran quality diagnostics. The provided samples passed the quality control test (on figure 3) as they clustered together with higher median intensities confirming their good quality [19]. Differentially-methylated loci were identified using single probe analysis implemented by our tool with the following parameters: phenotype set as **categorical** and qCutoff size set to **0.05**. The bump hunting algorithm was applied to identify DMRs with maximum location gap parameter set to **250**, genomic profile above the cutoff equal to **0.1**, number of resamples set to **0**, null method set to **permutation** and verbose equal **FALSE** which means that no additional progress information will be printed. Differentially-Methylated Regions and Positions revealed the need for further investigation of tissue diversity in response to environmental changes [26]. Nearest transcription start sites (TSS) found in the gene set can be listed as follows: PITX1, SFRP2, MSX1, MIR21, AXIN2, GREM1, WT1, CBX2, HCK, GTSE1, SNCG, PDPN, PDGFRA, NAF1, FGF5, FOXE1, THBS1, DLK1 and HOX gene family. The results of the re-analysis are available in the GitHub repository (https://github.com/kpbioteam/ewastools-case_study)

Important Findings

Although hyper-methylated genes identified by 'EWAS-suite' have been previously associated with cancer, this is the first time a link between them and MAPKi treatment resistance is reported. This data demonstrates that PDGFR, which is suggested to be responsible for RAS/MAPK pathway signaling. Trough activation may regulate the MAPKi mechanism in non responsive tumours. The methylation regulation of this altered status of PDGFR requires additional studies [25]. The PITX1 suppressor gene was found as one of the factors decreasing gene expression in human cutaneous malignant melanoma and might contribute to progression and resistance via promoting cell proliferative activity [27]. It has been found that the homeodomain transcription factor MSX1 and the CBX2 polycomb group protein are likely to be treatment resistance factors and are reported as downregulated and inactivated in melanoma tumours [28]. Previous published studies are limited to local surveys and serial biopsies. Thus, the stimulus of innate or acquired MAPKi resistance may be linked to epigenetics. GO annotation, provides information regarding the function of genes [29]. GO analysis identified :the pattern specification process (GO:0007389), skeletal system development (GO:0001501) and regionalisation (GO:000300) as significantly over-represented categories within the above DMR's, suggesting that melanoma MAPKi resistance could be related to the cells developmental process within specific environments.

Conclusion

With the rapidly increasing volume of epigenetics data available, computer-based analysis of heritable changes in gene expression becomes more and more feasible. Many genome-wide epigenetics studies have focused on generation of data, with data interpretation now being the challenge. Risk evaluation, disease management and novel therapeutic development are prompting researchers to find new bioinformatic frameworks and approaches. In this regard we propose a user friendly tool suite available via Galaxy platform. Ewastools allows life scientists to run complex epigenetics analysis. [16]. The case study presented provides a tangible example how the population epigenetics analysis can provide additional insights into melanoma therapeutic resistance.

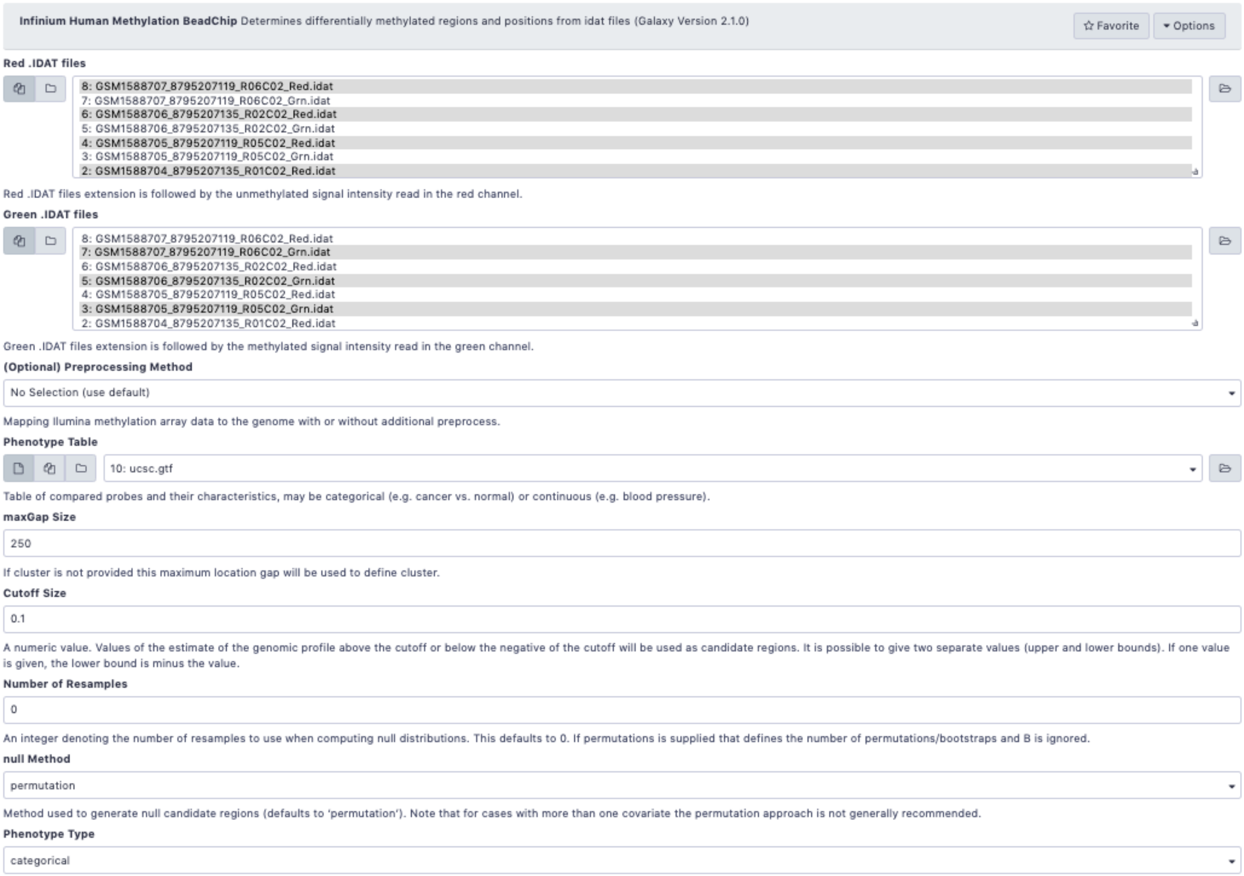

Figure 2. Screenshot from the Galaxy interface, showing Infinium Human Methylation BeadChip workflow as discussed in the analyses section.

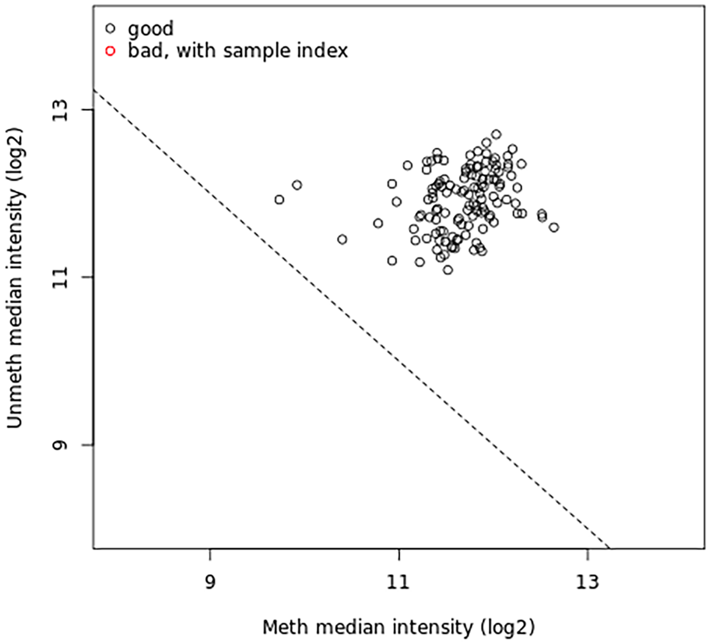

Figure 3. Quality control plot represent median intensity of melanoma pre and post MAPKi treatment samples. Plot compare median total intensity (Log2) of the methylated channel (x-axis) and unmethylated channel (y-axis). Bad-quality samples fall under the threshold and are colored red. There is no bad quality sample in this study.

Availability of source code and requirements

- Project name: Ewastools: Infinium Human Methylation BeadChip pipeline for population epigenetics integrated into Galaxy
- Project home page: https://github.com/kpbioteam/ewas_

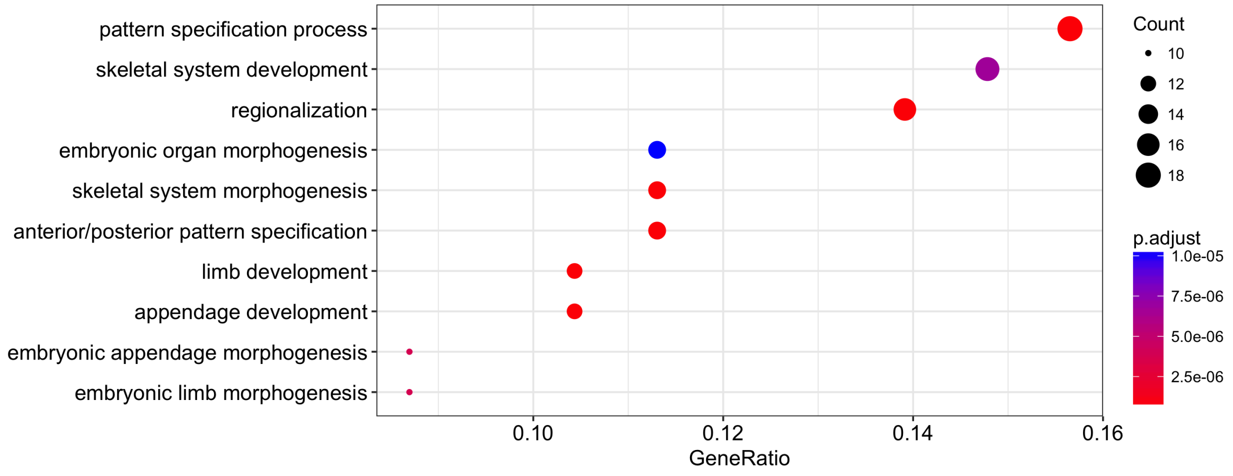

Figure 4. Functional Annotation of DMR's found in melanoma biopsies pre and post MAPKi treatment.

galaxy
- Operating system(s): Linux (recommended), Mac
- Programming language: R programming language (version 3.3.2, x86 64bit)
- Other requirements: Galaxy [22], Docker [30]
- License: MIT License
- biotoolsID identifier: biotools:ewastools
- RRID: SCR_018085

Availability of supporting data and materials

Test dataset in this article is available in the GEO database under accession GSE65186. The results of the re-analysis of the GSE65186 dataset are available in the GitHub repository (https://github.com/kpbioteam/ewastools-case_study). All tools described here are available in the Galaxy Toolshed (https://toolshed.g2.bx.psu.edu). The Dockerfile required to automatically deploy the pre-built Docker image is available at (https://galaxyproject.org/use/ewas-galaxy/).

List of Abbreviations

- DMP Differentially methylated positions
- DMR Differentially methylated regions
- EWAS Epigenome-wide association study
- GO Gene Ontology
- PCR Polymerase Chain Reaction
- RRBS Reduced Representation Bisulfite Sequencing
- SNP Single nucleotide polymorphism
- SNP Transcription Start Site
- WGBS Whole Genome Bisul-fite Sequencing

Competing interests

The authors declare that they have no competing interests.

Acknowledgements

We would like to thank Michal Gdula for constructive criticism of the manuscript.

References

1. Illumina I, Infinium Methylation Assay Overview; 2018. https//emea.illumina.com/science/technology/beadarray-technology/infinium-methylation-assay.html.
2. Lee JJ, Murphy GF, Lian CG. Melanoma epigenetics: novel mechanisms, markers, and medicines. Laboratory investigation 2014;94(8):822
3. Rakyan VK, Down TA, Balding DJ, Beck S. Epigenome-wide association studies for common human diseases. Nature Reviews Genetics 2011;12(8):529
4. Egger G, Liang G, Aparicio A, Jones PA. Epigenetics in human disease and prospects for epigenetic therapy. Nature 2004;429(6990):457
5. Klose RJ, Bird AP. Genomic DNA methylation: the mark and its mediators. Trends in biochemical sciences 2006;31(2):89–97
6. Sandoval J MSSMJPMBMEM Heyn H. Validation of a DNA methylation microarray for 450,000 CpG sites in the human genome. Epigenetics 2011;6(6):692–702.
7. Kristensen LS, Hansen LL. PCR based methods for detecting single locus DNA methylation biomarkers in cancer diagnostics, prognostics, and response to treatment. Clinical chemistry 2009;55(8):1471 1483 0009 9147.
8. Pidsley R, Wong CCY, Volta M, Lunnon K, Mill J, Schalkwyk LC. A data driven approach to preprocessing Illumina 450K methylation array data. BMC genomics 2013;14(1):293 1471 2164.
9. Marabita F, Almgren M, Lindholm ME, Ruhrmann S, Fagerström-Billai F, Jagodic M, et al. An evaluation of analysis pipelines for DNA methylation profiling using the Illumina HumanMethylation450 BeadChip platform. Epigenetics 2013;8(3):333–346
10. Peters TJ, Buckley M, Statham AL, Pidsley R, Clark SJ, Molloy PL. DMRcate Illumina 450 K methylation array apatial analysis methods. R package version 2014;1(0).
11. Hansen KD, Aryee M. minfi: Analyze Illumina's 450k methylation arrays. R package version 2012;1(0).
12. Morris TJ, Butcher LM, Feber A, Teschendorff AE, Chakravarthy AR, Wojdacz TK, et al. ChAMP 450k chip analysis methylation pipeline. Bioinformatics 2013;30(3):428 430 1460 2059.
13. Davis S, Du P, Bilke S, Triche T, Bootwalla M. methylumi Handle Illumina methylation data. R package version 2012;2(0).
14. Assenov Y, Muller F, Lutsik P, Walter J, Lengauer T, Bock

C. Comprehensive analysis of DNA methylation data with RnBeads. Nature methods 2014;11(11):1138 1548 7105.
15. Goecks J, Nekrutenko A, Taylor J. Galaxy: a comprehensive approach for supporting accessible, reproducible, and transparent computational research in the life sciences. Genome biology 2010;11(8):R86
16. Murat, Poterlowicz, Source Code of EWAS Tools; 2018. https//github.com/kpbioteam.
17. Murat, Poterlowicz, Published Tools; 2018. https//testtoolshed.g2.bx.psu.edu/repository/browse_repositories_in_categorysort=name&operation=repositories_by_user&id=0a77a6371a54a53.
18. Project G, Planemo documentation; 2014. https//planemo.readthedocs.io/en/latest/.
19. Aryee MJ, Jaffe AE, Corrada-Bravo H, Ladd-Acosta C, Feinberg AP, Hansen KD, et al. Minfi: a flexible and comprehensive Bioconductor package for the analysis of Infinium DNA methylation microarrays. Bioinformatics 2014;30(10):1363–1369
20. Jaffe AE, Murakami P, Lee H, Leek JT, Fallin MD, Feinberg AP, et al. Bump hunting to identify differentially methylated regions in epigenetic epidemiology studies. International journal of epidemiology 2012;41(1):200–209 1464–3685.
21. Consortium GO. The Gene Ontology (GO) database and informatics resource. Nucleic acids research 2004;32(suppl_1):D258–D261.
22. Murat, Poterlowicz, EWAS suite training; 2018. https://galaxyproject.github.io/training-material/topics/epigenetics/tutorials/ewas-suite/tutorial.html.
23. Murat, Poterlowicz, EWAS suite training data; 2018. https://zenodo.org/record/1251211#.WwREQ1Mvz-Y.
24. Verma M. Genome-wide association studies and epigenome-wide association studies go together in cancer control. Future Oncology 2016;12(13):1645–1664.
25. Hugo W, Shi H, Sun L, Piva M, Song C, Kong X, et al. Non genomic and immune evolution of melanoma acquiring MAPKi resistance. Cell 2015;162(6):1271 1285 0092 8674.
26. Bock C, Lengauer T. Computational epigenetics. Bioinformatics 2008;24(1):1 10 1367 4803.
27. Osaki M, Chinen H, Yoshida Y, Ohhira T, Sunamura N, Yamamoto O, et al. Decreased PITX1 gene expression in human cutaneous malignant melanoma and its clinicopathological significance. European Journal of Dermatology 2013;23(3):344 349 1167 1122.
28. Clermont PL, Sun L, Crea F, Thu KL, Zhang A, Parolia A, et al. Genotranscriptomic meta analysis of the Polycomb gene CBX2 in human cancers initial evidence of an oncogenic role. British journal of cancer 2014;111(8):1663 1532 1827.
29. Ashburner M, Ball CA, Blake JA, Botstein D, Butler H, Cherry JM, et al. Gene Ontology tool for the unification of biology. Nature genetics 2000;25(1):25 1546 1718.
30. Developers S, Docker documentation; 2017. https//media.readthedocs.org/pdf/docker-sean/latest/docker-sean.pdf.
