# Supplementary material for: Ewastools: Infinium Human Methylation BeadChip pipeline for population epigenetics integrated into Galaxy
Source: Gigascience. 2020 May 13;9(5):giaa049. doi: 10.1093/gigascience/giaa049 (PMC7219210; doi:10.1093/gigascience/giaa049)
Supplement: giaa049_Response_to_Reviewer_Comments_Original_Submission [file giaa049_response_to_reviewer_comments_original_submission.pdf]

Author's Response To Reviewer Comments

Close

We would like to thank the reviewers and the editors for their careful assessment of the manuscript and their detailed and constructive comments. We have extended the manuscript, clarified the methodologies and performed additional new experiments. Below we provide point-to-point responses with reference to the corresponding modifications of the manuscript. We believe that addressing these comments has produced a stronger manuscript that will hold more value for the scientific community.

#Editor Comments: In addition, please register any new software application in the bio.tools and SciCrunch.org databases to receive RRID (Research Resource Identification Initiative ID) and biotoolsID identifiers, and include these in your manuscript. This will facilitate tracking, reproducibility and re-use of your tool.

Authors:
Thank you for considering our revised manuscript. We have registered our tool in both bio.tools and in SciCrunch.org, and have provided the identifiers in our manuscript: biotoolsID identifier: biotools:ewastools, RRID: SCR_018085

Reviewer #1 Major concern:
The "Potential implications" section is confusing. It reads more of a use case or a proof of concept illustration of the value of the EWAS-Galaxy tools suite, rather than a section that discusses the "potential implications" for EWAS-Galaxy. I do like the idea of providing a use case, but I suggest renaming this section. I also suggest adding a little more background about the dataset being tested. Why was it chosen (the fact that there is "interest in skin cancer biomarker identification" doesn't seem like enough of a reason)? The dataset is published, which leads me to believe the authors are doing a re-analysis of the study. How do their results of identifying a set of DMRs/DMPs near transcription start sites and enhancers of the listed genes compare to what the original authors of the study found? I would love to see this use case expanded as I believe the goal is to highlight that EWAS-Galaxy can analyze (re-analyze?) methylation array data to drive hypothesis generation, which is an important point to make.
Authors:
We sincerely thank the reviewer for their thoughtful comments and inspections of our submission. We have renamed the section as suggested and provided more background about the dataset being tested (" …. Compared to genetic studies EWAS provides a unique opportunity to study dynamic response to treatment. It has been suggested that DNA methylation is associated with drug resistance.To validate our suite we have performed analysis of differentially-methylated regions using publicly available data from the Infinium Human Methylation BeadChip array of melanoma biopsies pre and post MAPKi treatment….”). The authors cannot agree more with the reviewer that it would be interesting to compare our results with what the original authors of the study found. Unfortunately the original study only provides a selected fragment of the results of the data analysis. Therefore we have created a dedicated GitHub repository https://github.com/kpbioteam/ewastools-case_study with the results of re-analysis of the original dataset.

Minor concerns:
Reviewer #1 comment#1
In the Background section the authors mention multiple open source software packages for analyzing methylation assay data (page 2, line 25). It appears that only Minfi tools made it into EWAS-Galaxy. It would be great if the authors mentioned whether there is ongoing work to incorporate these additional tools into EWAS-Galaxy or why only Minfi tools were included.
Authors:
We thank the reviewer for this comment. For now, we have focused on the implementation of the Minfi tools in the galaxy. We are happy to add additional tools if requested by users.
Reviewer #1 comment 2
The sentence starting "The tool suite includes methods…" on page 2, line 31 is weirdly worded. Bolded names of the tools are inserted into the sentence in a way that makes the sentence hard to read. The

same weird pattern is present on page 2 line 19. I would suggest re-wording these sentences to match the wording in the "Preprocessing and Normalization" and "Quality Assessment and Control" sections (where the bolded tool names make sense in the sentences).
Authors:
We agreed with this comment and changed the sentences accordingly
Reviewer #1 comment 3
There is mention of Illumina Genome Studio (page 2, line 20) before saying what it is (in Data Loading section). There is mention of Planemo (page 2, line 44) without mentioning or citing what it is. I would suggest describing these (and any other specialty) terms the first time they are mentioned.
Authors:
We agreed with this comment and changed the sentences accordingly
Reviewer #1 comment 4
I am unsure whether mentions of "Illumina Methylation Assay" (page 2, line 11), "450k assay" (page 2, line 14), "Infinium Methylation Assay" (page 2, line 48), and "Illumina 450k Methylation" (page 3, line 38) are all referring to the same assay type. I would suggest being consistent with naming or explicit about whether the different terms are the same assay type.
Authors:
We agreed with this comment and changed the sentences accordingly
Reviewer #1 comment 5
It is unclear what "bad, with sample index" means in the Figure 3 graph legend. Please clarify.
Authors:
We agreed with this comment and changed the sentences accordingly
Reviewer #1 comment 6
There is duplication of spelling out terms followed by the abbreviation in parentheses. In one example, "differentially-methylated regions (DMRs)" can be found three times in the text (page 2 line 62, page 3 line 35, and page 3 line 58). As per author instructions: "If abbreviations are used in the text they should be defined in the text at first use".
Authors:
We thank the reviewer for their careful inspection and modified accordingly
Reviewer #1 comment 7
An Abbreviations section is missing from the manuscript. As per author instructions: "a list of abbreviations should be provided in alphabetical order.".
Authors:
We agreed with this comment and provided list of abbreviations
Reviewer #1 comment 8
Figure numbering appears out of order. Figure 5 is called out before Figure 3. I do not see a call out to Figure 4. I also am not sure what conclusion I am supposed to draw from Figure 4. I suggest numbering and ordering the figures as they appear in the text and providing an explanation of what Figure 4 is showing.
Authors:
We thank the reviewer for pointing out this inconsistency, which has now been corrected.
Reviewer #1 comment 9
The Availability and requirements section is formatted strangely, and the section header includes "(Availability of source code and requirements (optional, if code is present))", which looks like it was copied from the author instructions and not removed. Please check formatting.
Authors:
We agreed with this comment and changed the section title and formatting accordingly
Reviewer #1 comment 10
There is mixed usage of US and UK English spelling (e.g. normalization and normalisation). Please standardize.
Authors:
We agreed with this comment and changed the spelling accordingly
Reviewer #2: Formatted review attached as PDF. Raw text is below.

Major concerns
Article Text
Reviewer #2 comment 1
This article, and especially the title, seem to indicate that the described tool suite is generally applicable to most/all population epigenetic study analysis.
However, the current implementation of this tool suite is limited to only handling Illumina Infinium methylation arrays, in particular the IlluminaHumanMethylation450 ('450k') array.
If your population epigenetic datasets are not from this specific technology iteration, then you cannot

use these Galaxy tool implementation to perform EWAS.
Either the article and title should be adjusted to accurately reflect the abilities of the described tools, or the tools should be updated to handle additional, including non-illumina methylation array, dataset types. This tool suite is currently several wrappers around minfi functions, that has been restricted to working with IlluminaHumanMethylation450kanno.ilmn12.hg19.
Authors:
We thank the reviewer for the comment. We have extended the tool suite to support: HumanMethylationEPIC and HumanMethylation27 methylation arrays. We fully understand the concerns of the reviewer around usability of the tool suite for population epigenetic datasets that are not from Illumina Infinium methylation arrays. However, at this moment the EWAS studies are predominantly generated using Illumina Infinium methylation arrays. EWAS Atlas (https://bigd.big.ac.cn/ewas) is one of the most comprehensive knowledge base of epigenome wide association studies and among the 1160 studies reported there we haven't found a single one that used a platform different than HumanMethylationEPIC (‘850k’) or IlluminaHumanMethylation450 ('450k') [accessed on 20/02/2020] .
We are happy to add additional tools if requested by users.
We have also adjusted the article title to “Ewastools: Infinium Human Methylation BeadChippipeline for population epigenetics integrated into Galaxy”

Reviewer #2 comment 2
Confirm that all tools e.g. listed in Table 1 are available in the ToolShed, Docker image, etc. (for example, the minfi_getanno does not exist, etc.)
Authors
We thank the reviewer for their careful inspection. We have not included Table 1 in the reviewed version of the manuscript as addressing one of the reviewer's comment below, the recent version of the suite consists a single ‘tool' with multiple functions.

Software
Reviewer #2 comment 3
Interoperability concerns. Stated purpose of toolset is to increase accessibility to EWAS methods, but toolset is very specific and not interoperable. 'Rdata' datatype is used as intermediate and end-pint datasets. Potential security concerns introduced by tools that consume untrusted serialized datasets, e.g. 'rdata' Rdata datasets cannot be used as inputs by standard Galaxy tools, so, essentially user gets 'locked in' to this pipeline implementation.
All outputs are a base 'rdata' datatype, despite containing different objects. This allows improper input/output dataset mixing. Hierarchical datatypes should be used.
Really, an intermediate filetype that is usable by other standard Galaxy tools (e.g. tabular files) would be advisable. At the very least, there should be tools to export to and from these specific binary types and more generalized formats.
Authors:

We agree with the reviewer and have modified the toolset so ‘Rdata’ datatype is no longer used.

Reviewer #2 comment 4
Majority of tools have an input for datasets and output of another. The interfaces do not allow configuring other options to the various functions.
Because intermediate datasets are rdata, and there are several steps that are just input file → output file (no other configurable), I have questions about the need to actually have them as separate steps. Does it make sense to have a 'tool' consist of multiple function calls instead?

Authors:
We agree with the reviewer and the recent version of the suite consists of a single tool with multiple functions.

Reviewer #2 comment 5
Minfi Map to Genome map to genome tool should assign dbkey to output dataset metadata in Galaxy
Tool should also allow selecting from a list of genomes/annotations
Would allow the other illumina arrays supported by minfi to work in this toolset
Authors:
We thank the reviewer for the comment. The recent version of the suite allows selecting different types of illumine arrays (>Genome Table option). We removed Minfi Map to Genome map to genome tool as the suite consists of a single tool with multiple functions.

Reviewer #2 comment 6
A "Galaxy Interactive Tour" is described as being available for the EWAS tools, but the tour.yaml file contains only a name and a description and not any actual tour content. (https://github.com/galaxyproject/training-material/blob/master/topics/epigenetics/tutorials/ewas-suite/tours/tour.yaml)

Authors:
We thank the reviewer for pointing out this inconsistency, which has now been corrected in the recent version of the training material https://github.com/galaxyproject/training-material/pull/1778

Reviewer #2 comment 7
Add license to https://github.com/kpbioteam/ewas_galaxy
Authors:
We thank the reviewer for the comment. We updated as requested.

Minor concerns
Reviewer #2 comment 8
Docker container is using Galaxy 18.05 and the pre-loaded EWAS tools are out-of-date
Should update to a newer version of Galaxy and update the contained EWAS-Galaxy tools. (docker run -it -p 8080:80 --rm kpbioteam/galaxy-ewas)
Logged in as an admin, and updated tools from ToolShed for the purpose of this review
Output of minfi dmr and minfi dmr tools creates an 'interval' type dataset. But these outputs have non-commented header lines, and cannot be displayed at UCSC as shown in tutorial without additional manual processing. This should be fixed at the tool output.
Authors:
We agree with the reviewer and have updated the docker container to Galaxy 19:09 and updated the contained tools from ToolShed. We have modified the tool to output 'dmr' and 'dmp' datasets as a 'bed' type
Reviewer #2 comment 9
The "Minfi Read 450k load .IDAT files" tool requires the datasets in the history to have names that have specific '_Red.idat' and '_Grn.idat' pattern matching. This is not explained in the tool, and if dataset names do not match, no warning or error is displayed to the user. This should be fixed as part of the tool.
Authors:
We thank the reviewer for the comment. We have explained now in the tool, the requirements for the datasets in the history to have names that have specific '_Red.idat' and '_Grn.idat' pattern matching i.e ("*Inputs*Series of .IDAT files: matching red and green .idat file for each sample on the chip intensity data"). This format follows the standard raw input for Illumina Methylation arrays as each sample have two files: one for red and green channels respectively.
Reviewer #2 comment 10
Authors:
Training material tutorial (https://training.galaxyproject.org/training-material/topics/epigenetics/tutorials/ewas-suite/tutorial.htm) has several errors, with described tool configurations/interfaces lacking the declared options, etc (e.g. minfi dmr tool, etc).
Also some missing steps (e.g. removing first lines of 'fake' interval files). Recommend walking through tutorial again and confirming all steps are listed and clear

We thank the reviewer for their careful inspection, which has now been corrected in the recent version of the training material https://github.com/galaxyproject/training-material/pull/1778.

Close
