# Supplementary material for: Ewastools: Infinium Human Methylation BeadChip pipeline for population epigenetics integrated into Galaxy
Source: Gigascience. 2020 May 13;9(5):giaa049. doi: 10.1093/gigascience/giaa049 (PMC7219210; doi:10.1093/gigascience/giaa049)
Supplement: giaa049_Response_to_Reviewer_Comments_Revision_1 [file giaa049_response_to_reviewer_comments_revision_1.pdf]

Author's Response To Reviewer Comments

Close

GIGA-D-19-00088R1
Ewastools: Infinium Human Methylation BeadChip pipeline for population epigenetics integrated into Galaxy
Katarzyna Murat; Björn Grüning; Paulina Wiktoria Poterlowicz; Gillian Westgate; Desmond J Tobin; Krzysztof Poterlowicz
GigaScience

Dear Dr Poterlowicz,

Your manuscript "Ewastools: Infinium Human Methylation BeadChip pipeline for population epigenetics integrated into Galaxy" (GIGA-D-19-00088R1) has been assessed by our reviewers. Based on these reports, and my own assessment as Editor, I am pleased to inform you that it is potentially acceptable for publication in GigaScience, once you have carried out some final edits that I will send along with an annotated version of the paper. The key one is to cite the GigaDB DOI in the Availability of Supporting Data section as follows:

Archival snapshots of the code are available in the GigaScience GigaDB repository [31].

Then you need to cite the DOI as reference 31 as follows:

31. Murat K; Gruening B; Poterlowicz PW; Westgate G; Tobin DJ; Poterlowicz K (2020): Supporting data for "Ewastools: Infinium Human Methylation BeadChip pipeline for population epigenetics integrated into Galaxy" GigaScience Database. http://dx.doi.org/10.5524/100744

Authors:

We would like to thank the reviewers and the editors for their careful assessment of the manuscript and we confirm that we addressed the comments accordingly.

Close
