# Supplementary material for: Ewastools: Infinium Human Methylation BeadChip pipeline for population epigenetics integrated into Galaxy
Source: Gigascience. 2020 May 13;9(5):giaa049. doi: 10.1093/gigascience/giaa049 (PMC7219210; doi:10.1093/gigascience/giaa049)
Supplement: giaa049_Reviewer_1_Report_Original_Submission — Mallory Freeberg, Ph.D. -- 4/10/2019 Reviewed [file giaa049_reviewer_1_report_original_submission.pdf]

Reviewer Report

Title: Ewastools: Infinium Human Methylation BeadChip pipeline for population epigenetics integrated into Galaxy

Version: Original Submission **Date:** 4/10/2019

Reviewer name: Mallory Freeberg, Ph.D.

Reviewer Comments to Author:

"EWAS-Galaxy: a tools suite for population epigenetics integrated into Galaxy" describes a suite of tools and a Galaxy workflow for analyzing methylation assay data to detect associations between epigenomic methylation patterns and population phenotypes. The tools are freely and openly available in the Galaxy Toolshed, and there is a Docker file available to launch a Galaxy environment to run the tools suite. It is clear the authors have put forth great effort to make their tools/workflow accessible to the scientific community, especially to non-programmers and those who might not have access to high-performance computing environments. I especially appreciate the authors' efforts to make and share Galaxy training materials. In general, I think EWAS-Galaxy would be a useful contribution to Galaxy and to the epigenomic community. I have some concerns about the manuscript that are organized below.
Major concern

1. The "Potential implications" section is confusing. It reads more of a use case or a proof of concept illustration of the value of the EWAS-Galaxy tools suite, rather than a section that discusses the "potential implications" for EWAS-Galaxy. I do like the idea of providing a use case, but I suggest renaming this section. I also suggest adding a little more background about the dataset being tested. Why was it chosen (the fact that there is "interest in skin cancer biomarker identification" doesn't seem like enough of a reason)? The dataset is published, which leads me to believe the authors are doing a re-analysis of the study. How do their results of identifying a set of DMRs/DMPs near transcription start sites and enhancers of the listed genes compare to what the original authors of the study found? I would love to see this use case expanded as I believe the goal is to highlight that EWAS-Galaxy can analyze (re-analyze?) methylation array data to drive hypothesis generation, which is an important point to make.

Minor concerns

1. In the Background section the authors mention multiple open source software packages for analyzing methylation assay data (page 2, line 25). It appears that only Minfi tools made it into EWAS-Galaxy. It would be great if the authors mentioned whether there is ongoing work to incorporate these additional tools into EWAS-Galaxy or why only Minfi tools were included.
2. The sentence starting "The tool suite includes methods…" on page 2, line 31 is weirdly worded. Bolded names of the tools are inserted into the sentence in a way that makes the sentence hard to read. The same weird pattern is present on page 2 line 19. I would suggest re-wording these sentences to match the wording in the "Preprocessing and Normalization" and "Quality Assessment and Control" sections (where the bolded tool names make sense in the sentences).
3. There is mention of Illumina Genome Studio (page 2, line 20) before saying what it is (in Data

Loading section). There is mention of Planemo (page 2, line 44) without mentioning or citing what it is. I would suggest describing these (and any other specialty) terms the first time they are mentioned.

4. I am unsure whether mentions of "Illumina Methylation Assay" (page 2, line 11), "450k assay" (page 2, line 14), "Infinium Methylation Assay" (page 2, line 48), and "Illumina 450k Methylation" (page 3, line 38) are all referring to the same assay type. I would suggest being consistent with naming or explicit about whether the different terms are the same assay type.
5. It is unclear what "bad, with sample index" means in the Figure 3 graph legend. Please clarify.
6. There is duplication of spelling out terms followed by the abbreviation in parentheses. In one example, "differentially-methylated regions (DMRs)" can be found three times in the text (page 2 line 62, page 3 line 35, and page 3 line 58). As per author instructions: "If abbreviations are used in the text they should be defined in the text at first use".
7. An Abbreviations section is missing from the manuscript. As per author instructions: "a list of abbreviations should be provided in alphabetical order.".
8. Figure numbering appears out of order. Figure 5 is called out before Figure 3. I do not see a call out to Figure 4. I also am not sure what conclusion I am supposed to draw from Figure 4. I suggest numbering and ordering the figures as they appear in the text and providing an explanation of what Figure 4 is showing.
9. The Availability and requirements section is formatted strangely, and the section header includes "(Availability of source code and requirements (optional, if code is present))", which looks like it was copied from the author instructions and not removed. Please check formatting.
10. There is mixed usage of US and UK English spelling (e.g. normalization and normalisation). Please standardize.

Level of Interest

Please indicate how interesting you found the manuscript: Choose an item.

Quality of Written English

Please indicate the quality of language in the manuscript: Choose an item.

Declaration of Competing Interests

Please complete a declaration of competing interests, considering the following questions:

- Have you in the past five years received reimbursements, fees, funding, or salary from an organisation that may in any way gain or lose financially from the publication of this manuscript, either now or in the future?
- Do you hold any stocks or shares in an organisation that may in any way gain or lose financially from the publication of this manuscript, either now or in the future?
- Do you hold or are you currently applying for any patents relating to the content of the manuscript?

- Have you received reimbursements, fees, funding, or salary from an organization that holds or has applied for patents relating to the content of the manuscript?
- Do you have any other financial competing interests?
- Do you have any non-financial competing interests in relation to this paper?

If you can answer no to all of the above, write 'I declare that I have no competing interests' below. If your reply is yes to any, please give details below.

I declare that I have no competing interests

I agree to the open peer review policy of the journal. I understand that my name will be included on my report to the authors and, if the manuscript is accepted for publication, my named report including any attachments I upload will be posted on the website along with the authors' responses. I agree for my report to be made available under an Open Access Creative Commons CC-BY license (http://creativecommons.org/licenses/by/4.0/). I understand that any comments which I do not wish to be included in my named report can be included as confidential comments to the editors, which will not be published.

Choose an item.

To further support our reviewers, we have joined with Publons, where you can gain additional credit to further highlight your hard work (see: https://publons.com/journal/530/gigascience). On publication of this paper, your review will be automatically added to Publons, you can then choose whether or not to claim your Publons credit. I understand this statement.

Yes Choose an item.
