# Supplementary material for: Ewastools: Infinium Human Methylation BeadChip pipeline for population epigenetics integrated into Galaxy
Source: Gigascience. 2020 May 13;9(5):giaa049. doi: 10.1093/gigascience/giaa049 (PMC7219210; doi:10.1093/gigascience/giaa049)
Supplement: giaa049_Reviewer_2_Report_Original_Submission — Daniel Blankenberg -- 10/28/2019 Reviewed [file giaa049_reviewer_2_report_original_submission.pdf]

Reviewer Report

Title: Ewastools: Infinium Human Methylation BeadChip pipeline for population epigenetics integrated into Galaxy

Version: Original Submission **Date:** 10/28/2019

Reviewer name: Daniel Blankenberg

Reviewer Comments to Author:

Formatted review attached as PDF. Raw text is below.
Summary
This article reports on "EWAS-Galaxy: a tools suite for population epigenetics integrated into Galaxy". Which aims to make tools for performing Epigenome-wide association study analysis more flexible and accessible to everyone via a Galaxy implementation including training materials.
Review
Review was performed primarily using the provided Docker container, although local installation from the ToolShed was briefly tested.
In addition to several usability concerns, as outlined below, I would very much like to see the tool suite expanded to encompass generalized population epigenetics studies (GWAS) as implied by the title, or the specific nature (IlluminaHumanMethylation450 array on hg19) of the tool suite more accurately expressed.
Major concerns
Article Text
This article, and especially the title, seem to indicate that the described tool suite is generally applicable to most/all population epigenetic study analysis.
However, the current implementation of this tool suite is limited to only handling Illumina Infinium methylation arrays, in particular the IlluminaHumanMethylation450 ('450k') array.
If your population epigenetic datasets are not from this specific technology iteration, then you cannot use these Galaxy tool implementation to perform EWAS.
Either the article and title should be adjusted to accurately reflect the abilities of the described tools, or the tools should be updated to handle additional, including non-illumina methylation array, dataset types.
This tool suite is currently several wrappers around minfi functions, that has been restricted to working with IlluminaHumanMethylation450kanno.ilmn12.hg19.
Confirm that all tools e.g. listed in Table 1 are available in the ToolShed, Docker image, etc. (for example, the minfi_getanno does not exist, etc.)
Software
Interoperability concerns. Stated purpose of toolset is to increase accessibility to EWAS methods, but toolset is very specific and not interoperable.
'Rdata' datatype is used as intermediate and end-pint datasets
Potential security concerns introduced by tools that consume untrusted serialized datasets, e.g. 'rdata'

Rdata datasets cannot be used as inputs by standard Galaxy tools, so, essentially user gets 'locked in' to this pipeline implementation.
All outputs are a base 'rdata' datatype, despite containing different objects. This allows improper input/output dataset mixing.
Hierarchical datatypes should be used.
Really, an intermediate filetype that is usable by other standard Galaxy tools (e.g. tabular files) would be advisable.
At the very least, there should be tools to export to and from these specific binary types and more generalized formats.
Majority of tools have an input for datasets and output of another. The interfaces do not allow configuring other options to the various functions.
Because intermediate datasets are rdata, and there are several steps that are just input file â†’ output file (no other configurable), I have questions about the need to actually have them as separate steps.
Does it make sense to have a 'tool' consist of multiple function calls instead?
Minfi Map to Genome map to genome tool should assign dbkey to output dataset metadata in Galaxy
Tool should also allow selecting from a list of genomes/annotations
Would allow the other illumina arrays supported by minfi to work in this toolset
A "Galaxy Interactive Tour" is described as being available for the EWAS tools, but the tour.yaml file contains only a name and a description and not any actual tour content.
(https://github.com/galaxyproject/training-material/blob/master/topics/epigenetics/tutorials/ewas-suite/tours/tour.yaml)
Add license to https://github.com/kpbioteam/ewas_galaxy
Minor concerns
Docker container is using Galaxy 18.05 and the pre-loaded EWAS tools are out-of-date
Should update to a newer version of Galaxy and update the contained EWAS-Galaxy tools. (docker run -it -p 8080:80 --rm kpbioteam/galaxy-ewas)
Logged in as an admin, and updated tools from ToolShed for the purpose of this review
Output of minfi dmr and minfi dmr tools creates an 'interval' type dataset. But these outputs have non-commented header lines, and cannot be displayed at UCSC as shown in tutorial without additional manual processing. This should be fixed at the tool output.
The "Minfi Read 450k load .IDAT files" tool requires the datasets in the history to have names that have specific '_Red.idat' and '_Grn.idat' pattern matching. This is not explained in the tool, and if dataset names do not match, no warning or error is displayed to the user. This should be fixed as part of the tool.
Training material tutorial (https://training.galaxyproject.org/training-material/topics/epigenetics/tutorials/ewas-suite/tutorial.htm) has several errors, with described tool configurations/interfaces lacking the declared options, etc (e.g. minfi dmr tool, etc).
Also some missing steps (e.g. removing first lines of 'fake' interval files). Recommend walking through tutorial again and confirming all steps are listed and clear.

Level of Interest

Please indicate how interesting you found the manuscript: Choose an item.

Quality of Written English

Please indicate the quality of language in the manuscript: Choose an item.

Declaration of Competing Interests

Please complete a declaration of competing interests, considering the following questions:

- Have you in the past five years received reimbursements, fees, funding, or salary from an organisation that may in any way gain or lose financially from the publication of this manuscript, either now or in the future?
- Do you hold any stocks or shares in an organisation that may in any way gain or lose financially from the publication of this manuscript, either now or in the future?
- Do you hold or are you currently applying for any patents relating to the content of the manuscript?
- Have you received reimbursements, fees, funding, or salary from an organization that holds or has applied for patents relating to the content of the manuscript?
- Do you have any other financial competing interests?
- Do you have any non-financial competing interests in relation to this paper?

If you can answer no to all of the above, write 'I declare that I have no competing interests' below. If your reply is yes to any, please give details below.

I declare that I have no competing interests

I agree to the open peer review policy of the journal. I understand that my name will be included on my report to the authors and, if the manuscript is accepted for publication, my named report including any attachments I upload will be posted on the website along with the authors' responses. I agree for my report to be made available under an Open Access Creative Commons CC-BY license (http://creativecommons.org/licenses/by/4.0/). I understand that any comments which I do not wish to be included in my named report can be included as confidential comments to the editors, which will not be published.

Choose an item.

To further support our reviewers, we have joined with Publons, where you can gain additional credit to further highlight your hard work (see: https://publons.com/journal/530/gigascience). On publication of this paper, your review will be automatically added to Publons, you can then choose whether or not to claim your Publons credit. I understand this statement.

Yes Choose an item.
